# Mentoring stages: A study of undergraduate mentoring in palliative medicine in Singapore

**DOI:** 10.1371/journal.pone.0214643

**Published:** 2019-04-24

**Authors:** Lalit Krishna, Ying Pin Toh, Stephen Mason, Ravindran Kanesvaran

**Affiliations:** 1 Palliative Care Institute Liverpool, Academic Palliative & End of Life Care Centre, University of Liverpool, Liverpool, United Kingdom; 2 Division of Palliative and Supportive Care, National Cancer Centre Singapore, Singapore, Singapore; 3 Yong Loo Lin School of Medicine, National University of Singapore, Singapore, Singapore; 4 Centre of Biomedical Ethics, National University of Singapore, Singapore, Singapore; 5 Duke- NUS Medical School, Singapore, Singapore; 6 National University Hospital Singapore, Department of Family Medicine, Singapore, Singapore; 7 Division of Medical Oncology, National Cancer Centre Singapore, Singapore, Singapore; Berner Fachhochschule, SWITZERLAND

## Abstract

**Background:**

Mentoring nurtures a mentee’s personal and professional development. Yet conflation of mentoring approaches and a failure to contend with mentoring’s nature makes it difficult to study mentoring processes and relationships. This study aims to understand of mentee experiences in the Palliative Medicine Initiative (PMI). The PMI uses a consistent mentoring approach amongst a homogeneous mentee population offers a unique opportunity to circumnavigate conflation of practices and the limitations posed by mentoring’s nature. The data will advance understanding of mentoring processes.

**Methods:**

Sixteen mentees discussed their PMI experiences in individual face-to-face audio-recorded interviews. The two themes identified from thematic analysis of interview transcripts were the stages of mentoring and communication.

**Results:**

The 6 stages of mentoring are the ‘pre-mentoring stage’, ‘initial research meetings’, ‘data gathering’, ‘review of initial findings, ‘manuscript preparation” and ‘reflections’. These subthemes sketch the progression of mentees from being dependent on the mentor for support and guidance, to an independent learner with capacity and willingness to mentor others. Each subtheme is described as stages in the mentoring process (mentoring stages) given their association with a specific phase of the research process.

Mentoring processes also pivot on effective communication which are influenced by the mentor’s characteristics and the nature of mentoring interactions.

**Conclusion:**

Mentoring relationships evolve in stages to ensure particular competencies are met before mentees progress to the next part of their mentoring process. Progress is dependent upon effective communication and support from the mentor and appropriate and timely adaptations to the mentoring approach to meet the mentee’s needs and goals. Adaptations to the mentoring structure are informed by effective and holistic evaluation of the mentoring process and the mentor’s and mentee’s abilities, goals and situations. These findings underline the need to review and redesign the way assessments of the mentoring process are constructed and how mentoring programs are structured.

## Introduction

### Background

Mentoring in Palliative Medicine has been shown to enhance clinical skills [1–5], inculcate appropriate “attitudes and practices in caring for dying patients” [6], increase research output (7–10) and boost the reputation of host organizations [7–10]. Yet these gains have not been accompanied by meaningful advancements in the understanding of the mentoring process nor in the mentoring relationships that underpin mentoring’s success [7–10]. These gaps in understanding have been attributed to the conflation of distinct forms of mentoring such as near-peer, peer, group, mosaic, leadership, patient, family, youth, and e-mentoring and the mistaken intermixing of mentoring with role modelling, supervision, advising, tutoring, sponsoring, networking, teaching, counselling and/or coaching in many studies [10–17]. The viability of prevailing concepts of mentoring are also called into question by the failure of many studies to account for mentoring’s evolving, goal-specific, context-sensitive, mentor-, mentee-, organizational- and relational-dependent nature (henceforth mentoring’s nature) which limits studies of mentoring practices to programs with similar approaches, goals and mentee and mentor populations [10–19]. These considerations call into question the validity of the theories of mentoring shaped by prevailing studies.

Seeking to better understand “what are the experiences of mentees in a novice mentoring program?” we proposed a semi-structured interview study of the Palliative Medicine Initiative (PMI) program. With concepts of mentoring open to conjecture, the study of mentee experiences in the PMI offers a unique opportunity to understand the mentoring process and interactions in a program that employs a consistent and well-defined novice mentoring approach involving a clearly delineated mentor and mentee population. Design of the PMI was informed by prevailing accounts of novice mentoring. Defined as a “*dynamic*, *context dependent*, *goal sensitive*, *mutually beneficial relationship between an experienced clinician (mentor) and junior clinicians and/or undergraduates (mentee) that is focused upon advancing the development of the mentee*” the PMI’s novice mentoring circumnavigates the limitations posed by mentoring’s nature and conflation of mentoring practices and provides the basis for the PMI’s novice mentoring approach [8] (Table 1).

**Table 1:** The PMI novice mentoring approach.

The Palliative Medicine Initiative (PMI) Novice Mentoring approach
1. All mentors recruited to the PMI were experienced clinicians and of consultant or attending grade at the Department of Palliative Medicine (DPM) at the National Cancer Centre Singapore (NCCS) and were registered Palliative Medicine specialists with the Singapore Medical Council. 2. All mentors were provided with mentor training to ensure a consistent novice mentoring approach [20–22]. 3. Mentee-initiated matching was promoted to create enduring and personalized relationships [23–25]. The PMI offered all medical students the opportunity to initiate one-to-one mentoring relationships with one of 6 PMI mentors at DPM during their 2-week Palliative Medicine electives. 4. Medical students were given the opportunity to work with mentors of the same gender and background in keeping with prevailing reports that this improved mentoring outcomes [25–28]. 5. Mentees were briefed on the professional, mentoring and research interests of the 4 female and 2 male PMI mentors and provided guidance on how to select a mentor. 6. PMI mentees were also informed of a mentor’s and mentee’s roles and responsibilities and briefed on the PMI’s individual face-to-face, dyadic mentoring approach to better prepare them for the PMI mentoring process [21, 23, 29–33]. 7. As with other mentoring programs at the time, mentees who had selected a mentor, were invited to attend pre-mentoring meetings where mentees and mentors discussed potential research topics, their aspirations, expectations and concerns and established the goals, timelines, roles and responsibilities, expectations, codes of conduct and the frequency of face-to-face meetings [34–36]. 8. Unlike most programs the PMI program could not provide mentees with ‘protected time’. Most of the mentoring process within the PMI took place in the mentee’s spare time with only two weeks of their involvement in the PMI given academic recognition by the university. 9. PMI mentors were provided with ‘protected time’ to pursue their education and mentoring projects [37]. 10. As with many of the prevailing programs at the time mentor contributions to the PMI program and mentoring successes were considered in their yearly appraisals and in applications for promotion and academic credentialing [22, 24, 38–40]. PMI mentors were also given priority for funding and leave for education meetings and conferences.

Study of PMI mentee experiences provides an opportunity to study key characteristics of mentoring relationships in a program that has supported more than 40 solo, mentee co-authored and/or first authored mentee publications in peer reviewed journals and more than 50 posters at international conferences in Palliative Medicine, medical ethics, medical education, End-of-life Ethics (EoLE) and Health Services Research over the last 7 years. These insights will help design and marshal support and oversight of mentoring programs [3, 5, 20, 26, 30, 35, 36, 38, 41–65].

## Methodology

In the absence of an *a priori* framework of mentoring [66–70] and data suggesting that novice mentoring is a ‘social phenomena’, a qualitative approach was adopted to examine the individual experiences and views of mentees [7–11, 13, 15–17, 58, 71, 72]. A qualitative approach also addresses the issue of an “*over-reliance on cross-sectional designs and self-report data*, *a failure to differentiate between different forms of mentoring (e*.*g*., *formal versus informal)*, *lack of dyadic data*, *and the use of psychometrically questionable measures*” that fail to fully consider the impact of mentoring’s evolving nature [29]. To provide a holistic perspective of the mentoring experience and preserve *“the participant’s voice and meaning present in the theoretical outcome”*, face-to-face semi-structured interviews with individual mentees were adopted [29]. Individual face-to-face semi-structured interviews with mentees also provide a platform for “*a rich and detailed exploration*” of the motives, views and experiences of mentees at different phases of their mentoring journey whilst allowing interviewers to delve deeper into various responses and provide a means of “*pursuing additional relevant topics as they arise*” [71].

The time-frame selected for study was 2013–2015 in order to capture the effects of changes made to the program following the untimely death of one of the founding members of the program and the introduction of a structured mentor training program, formal mentee briefings by senior mentors and pre-mentoring meetings to better prepare potential mentees and instill greater consistency within the mentoring process. Total sampling was used to recruit all 16 mentees who had completed their PMI projects between 2013–2015. There were no dropouts during this period. There were 6 male and 10 female medical students who began their participation in the PMI in year 2 and 3 of a 5-year undergraduate medical program at NUS.

### Ethics

The PMI Review was approved by the Central Institutional Review Board of the National Cancer Centre Singapore (CIRB Ref 2015/2281: Evaluation of the Palliative Medicine Initiative (PMI) Mentorship Program). All participants provided written informed consent.

Funding for the transcription of the audio-recorded interviews came from an Academic Medicine Education Institute (AMEI) Duke-NUS Innovation Grant (EING1508).

### Semi-structured interviews

The semi-structured questionnaire was designed upon data drawn from systematic reviews of novice mentoring relationships, programs and prevailing tools [7–11, 13, 15–17, 58, 71, 72]. The draft questions were then reviewed by PMI stakeholders, local Palliative Medicine experts and educationalists and the refined semi-structured questionnaire was piloted amongst PMI mentees who did not meet the inclusion criteria for this study. This process ensured that the semi-structured questionnaire was methodologically and culturally appropriate to the local setting.

An experienced interviewer who has participated in the PMI in 2011 was trained to conduct the interviews. The interviewer had no associations with the interviewees. The interviewer’s experience in the PMI allowed a deeper understanding of the mentee’s responses and the nonverbal cues noted during the interviews. The interviewer was also provided with feedback from the two independent reviewers analyzing the interview transcripts as part of the iterative process employed as part of this study.

All 16 participants completed the interviews within an hour, with the average interview lasting 47 minutes. Transcription of the audio-records was carried out by a private transcription service. The transcripts were then anonymized by the interviewer and verified by the interviewees before being dispatched for analysis.

### Analysis of the transcripts

Three reviewers, experienced in the use of Braun and Clarke’s approach to thematic analysis carried out independent analyses of the anonymised transcripts [73]. To enhance rigour, analyses of the interview transcripts focused upon descriptions of PMI experiences to preserve the participant’s ‘voice’ and the integrity of their ideas, emotions and beliefs. An inductive approach allowed themes to be “inductively defined from the raw data without any predetermined classification”[74].

In keeping with the first phase of Braun and Clarke’s approach, an iterative step-by-step thematic analysis was carried out with the first 10 anonymised transcripts. The three researchers (LK, TYP and RK) ‘actively’ read the transcripts several times to familiarise themselves with the information. By immersing themselves in the data and making notes, the reviewers sought to meaning and patterns in the data.

Next, the reviewers constructed ‘codes’ from the ‘surface’ meaning of the mentee’s responses contained within the three interview transcripts. Braun and Clarke describe a code as a “feature of the data (semantic content or latent) that appears interesting to the analyst, and refer to ‘the most basic segment, or element, of the raw data or information that can be assessed in a meaningful way regarding the phenomenon’[75]. Unlike Braun and Clarke’s original description the reviewers used Excel forms to capture the initial codes. The reviewers also created notes to explain what the codes were and the reviewers thoughts about them. The initial codes from ‘open coding’ were then grouped into categories according to their similarities.

In the third phase of Braun and Clarke’s approach categories were organised into themes that best depict the data [73]. Two reviewers [LK and TYP] used mind maps to illustrate the links between the various codes and to help delineate themes whilst the other reviewer employed lists (RK) to identify potential themes. Braun and Clarke describe themes as “something important about the data… and represents some level of patterned response or meaning within the data set”.

Each reviewer reviewed and refined their themes to ensure they were first coherent and then representative of the whole data set. The reviewers carried out between three to five refinements of the themes in this fourth phase of Braun and Clarke’s approach.

In this fifth phase of Braun and Clarke’s approach, the reviewers continued to work independently naming and delineating the specific characteristics of each theme. The three reviewers reported no new themes after the first 8 transcripts. Once the themes were established, the reviewers proceeded to the final phase in creating a report of their analysis of the 10 transcripts analysed and discussed them in online and face-to-face meetings. The three reviewers then compared their findings and agreed upon a common coding framework and code book using Sambunjak, Straus & Marusic’s [58] “negotiated consensual validation” approach. The code book consisted of the codes, sub-themes, definitions, descriptions of terms and guidelines on when to use and when not to apply [76].

In using the code book to code and analyses the rest of the transcripts, the reviewers maintained an iterative approach to the analysis. The three reviewers coded the rest of the transcripts independently using the common coding framework and code book and grouped the ‘detail rich’ codes together to identify larger inclusive concepts. As new codes emerged, these were associated with previous codes and concepts [77]. The codes and the larger inclusive concepts were collapsed into themes and subthemes [78]. The coding framework and code book were reviewed as part of the iterative process employed by this study. The reviewers independently extracted and classified all quotations under each theme.

An external reviewer analyzed the quotes assigned to each code for consistency and accuracy [78]. The themes identified by each reviewer were discussed online and at an author’s meeting where consensus on a final list of themes and subthemes was achieved using the “negotiated consensual validation” approach. The themes and subthemes were reviewed once more to ensure that the themes and the respective quotes mapped to experiences with the PMI processes, mentor and mentee related issues, mentoring relationships, organizational factors and environmental influences.

To ensure the robustness of the analysis a detailed description of the results replete with quotations that illustrated the descriptions of each theme was reviewed by the external reviewer.

### Validity and reliability of the analysis

For the purposes of triangulation, the analysis was carried out by three independent reviewers. The codes and themes identified by each reviewer was discussed by the three reviewers in online and face-to-face meetings. In addition, the findings of each reviewer and their combined analysis was reviewed by an experienced external reviewer well versed in the topic at hand. To further ensure theoretical validation the results of the analysis was compared with prevailing data. An iterative process was employed which meant that any new codes identified meant that all the transcripts were reviewed to verify the classification and ensure complete data extraction.

## Results

Thematic analysis of the transcripts revealed 2 themes including ‘stages of mentoring’ and ‘communication’. The ‘stages of mentoring’ contained ‘time bound subtheme’ that meant that the events captured occurred at different points of the experience [74]. ‘Communication’ contained ‘transversal subthemes’ that spanned the length of the mentoring process. We discuss each in turn.

### Stages of mentoring

‘Pre-mentoring stage’, ‘initial research meetings’, ‘data gathering’, ‘review of initial findings, ‘manuscript preparation” and ‘reflections’ are the 6 subthemes delineated. These subthemes sketch the progression of mentees from being dependent on the mentor for support and guidance, to an independent learner with capacity and willingness to mentor others. Each subtheme is described as stages in the mentoring process (mentoring stages) given their association with a specific phase of the research process. To contextualize the mentoring stages a brief description of each phase of the research process associated with it is necessary.

#### i. Pre-mentoring stage

The pre-mentoring stage involves the mentee’s initiating a mentoring relationship with the PMI mentor of their choosing. All mentees reported selecting a mentor based on the information provided about each PMI mentor and the mentee’s experiences working with the mentor. Mentees report a variety of emotions during this stage of the mentoring process.

“At the beginning I was a bit lost” (Mentee 1)

This was contributed to by gaps in knowledge and inexperience in the matching and research process.

*“I didn't really understand what was going on*, *and what we're supposed to get.” (Mentee 5)*

#### ii. Initial research meetings

The initial research meetings provide mentees with an opportunity to shape the research question, project goals, timelines, the frequency of meeting and the roles and responsibilities of the mentee and the mentor, learn about the research process and establish the expectations upon them. Initial research meetings also provide mentors with a chance to assess and personalize their mentoring approach to account for the mentee’s abilities, needs and timelines.

*“*..*right at the start*, *he drew out like a mind map*, *kind of picture*, *explaining the process and what and how we should do it*.” *(Mentee 2)*

#### iii. Data gathering

The data gathering stage includes initiation of the research program and the data collection phase. Pivotal to this process is personalization of the mentoring approach through evaluation of the mentee’s abilities and needs. Mentoring processes were adapted to the

- Mentee’s situation.
  *“During times of high workload from school or when there were family or personal psychosocial issues I was struggling with*, *[my mentor] would readily take the research load off me so that I could settle all these issues without having to worry about the research tasks*.” *(Mentee 7)*
- Mentor’s evaluation of the mentee’s abilities.
  *“When we first started*, *Dr X’s instructions seemed to be targeted to a higher level*. *But when Dr X realized that me and my colleague were doing this for the first time Dr X tailored the guidance to our level*.” *(Mentee 10)*

#### iv. Review of study findings

This stage saw mentees take on more responsibilities and ‘ownership of the process’ as they analyzed the data accrued and discussed the findings with the mentor.

*“The mentor assigned me research roles and tasks that are suited for my level of experience*. *Over time these tasks required more reflection and analysis.” (Mentee 3)*

#### v. Manuscript preparation

Manuscript and/or poster preparation motivated mentees.

*“… as we went along, I realized that this was something real*. *I can get something out of it. I can churn out paper. I can do a poster, I can present at some conference.” (Mentee 13)*

#### vi. Reflections and aspirations

Mentee reflections following the completion of the PMI projects focused upon appreciation of different aspects of clinical care and new aspirations.

- Appreciation of clinical care
  - Recognizing the skills needed
    *“medicine is* ..*an art-form.” (Mentee 9)*
    Other mentees described learning the importance of being sensitive to non-verbal communication and the need to listen to the perspectives of patients and their families.
  - Sociocultural aspects
    “My mentor brought extra perspective in terms of social cultural factors and how to actually balance patient goals with clinical goals” (Mentee 11)
  - Patient-centered care
    *“It has helped me become more sensitive to the reactions of patients for example*.” *(Mentee 4)*
    Realizing the need to understand the patient as a person was a common outcome which several mentees described.
  - Holistic care
    *“[the mentoring process has] helped me to be more open-minded and understanding in clinical practice, I’ve learnt to see things from various angles*.*” (Mentee 6)*
    Another mentee described how the process changed her approach to patient care to orientated around patient’s goals and priorities.
  - Greater appreciation of research
    *“Research does seem more meaningful now*, *like*, *they actually have significant impact on medical practice and patient care*.” *(Mentee 7)*
- Aspirations
  - ‘Pay it forward’
    *“I used to think that I’ll never ever*, *ever want to teach students*. *But I think after going through Dr X*. *I actually wouldn’t mind mentoring a few students ‘cause I think it provides a positive effect on someone’s life too*.” *(Mentee 5)*
    Mentees expressed the desire to commit to mentoring others in future, realizing how mentoring can shape the future of medicine.
  - Aspiration to be effective educators and mentors in future.
    *“..now I find myself thinking of new ideas*, *and thinking of new potential projects that I could undertake*” *(Mentee 16)*
    Another mentee discussed around mentoring styles, and the need for mentors to respect the mentee and be interested in their well-being in order to be effective mentors.
  - Aspire to be good clinicians
    *“[the program] showed me that I could do much more for patients as a physician than I had previously thought”* (Mentee 14)
    Mentees describing learning from mentors perspective taking and the importance of honoring the humanity of patients as one interacts with them.

### Communication

The three subthemes delineated under this theme were the characteristics of mentors, the nature of communication and the obstacles to mentee-mentor interactions.

- Characteristics of mentor influenced communication. These include
  - Welcoming
    “I felt welcomed as a colleague and a fellow investigator” (Mentee 8)
    Mentees described feeling a sense of belonging to the mentoring team and felt valued as a team member.
  - Concern
    “My mentor genuinely cared about our well-being” (Mentee 16)
    Several mentees expressed that mentors’ constant enquires about their well-being helped mentees to feel supported.
  - Motivating
    *“..you can see that he’s very enthusiastic. The enthusiasm you know*, *it rubs off” (Mentee 13)*
    Mentees described that the mentors’ consistently encouraged and motivated them to persevere on to complete their projects.
  - Individualized
    *“If my psychosocial issues outside of research weren’t settled*, *and on not too few occasions without my mentor’s help*, *I would have given up on the projects long ago*.*” (Mentee 11)*
    Other mentees described how the mentors were understanding when they experienced other life stressors and how help was rendered during these periods to lighten their workload.
  - Encouraging
    *“He encourages you*, *he doesn’t pressurize you” (Mentee 13)*
    Several mentees expressed appreciating the mentors’ affirmation and positive feedback which validated their efforts. Mentees also shared that the mentors’ provided much needed moral support through the mentoring process.
  - Constructive
    *“..it was very important to have the feedback*, *so that I can do it better*”*(Mentee 8)*
    Several mentees emphasized the importance of feedback as they developed through the mentoring process.
- The nature of mentor-mentee interactions revealed a number of unique features
  - Evolved from a formal hierarchical to a more casual style
    *“My text[s] became less formal* … *it becomes a lot easier to ask questions” (Mentee 15)*
    *“…slowly it becomes more comfortable*. *Cause at first, we don’t really know each other. After that we became friends” (Mentee 10)*
  - Reciprocal, where feedback from mentees shaped the mentoring approach
    *‘When the mentor realized that*, *it’s the first time doing this, he tailored things to my level’ …the [mentor’s] feedback allowed me to gain confidence” (Mentee 7)*
  - Responsive
    Nearly all mentees were anxious at each new stage of the mentoring process. *“I could get a little bit stressed*, *‘cause I didn’t know what to do” (Mentee 13)*.
    These concerns were overcome by specific guidance and personalized support provided. *“My mentor’s guidance made me feel more confident in contributing to the paper and have a greater sense of ownership over the project*.*” (Mentee 1)*
  - A sense of professional ‘distance’ remained
    *“My mentor managed to maintain a professional enough relationship, without becoming too friendly*, *which can sometimes be detrimental for the mentoring process” (Mentee 5)*
  - Sounding board for the mentee’s new concepts and proposals.
    *“I also liked how he is open to hearing my random ideas… basically I bounce ideas off him*, *which helps me learn a lot really” (Mentee 3)*
    Other mentees described a non-judgmental platforms where they were free to express their ideas.
- Obstacles to mentee-mentor interactions pivot on poor communication and feedback and a lack of organizational support for the mentoring process. Poor communication and feedback saw mentees struggle to comprehend the tasks assigned to them and the expectations upon them leading to poor mentoring relationships.
  *“The mentor’s instructions were targeted at someone at higher level of competency rather than a novice*” (Mentee 13)
  It also saw mentees intimidated by the views and had difficulty expressing their own ideas.
  *“I still felt that I’m not good enough to contribute”* (Mentee 8)
  A lack of protected time and a lack of recognition of a student’s participation in the mentoring process threatened the mentoring process. Mentees cited a lack of time and competing commitments as key obstacles to effective participation in the PMI program.

## Discussion

**Fig 1:**
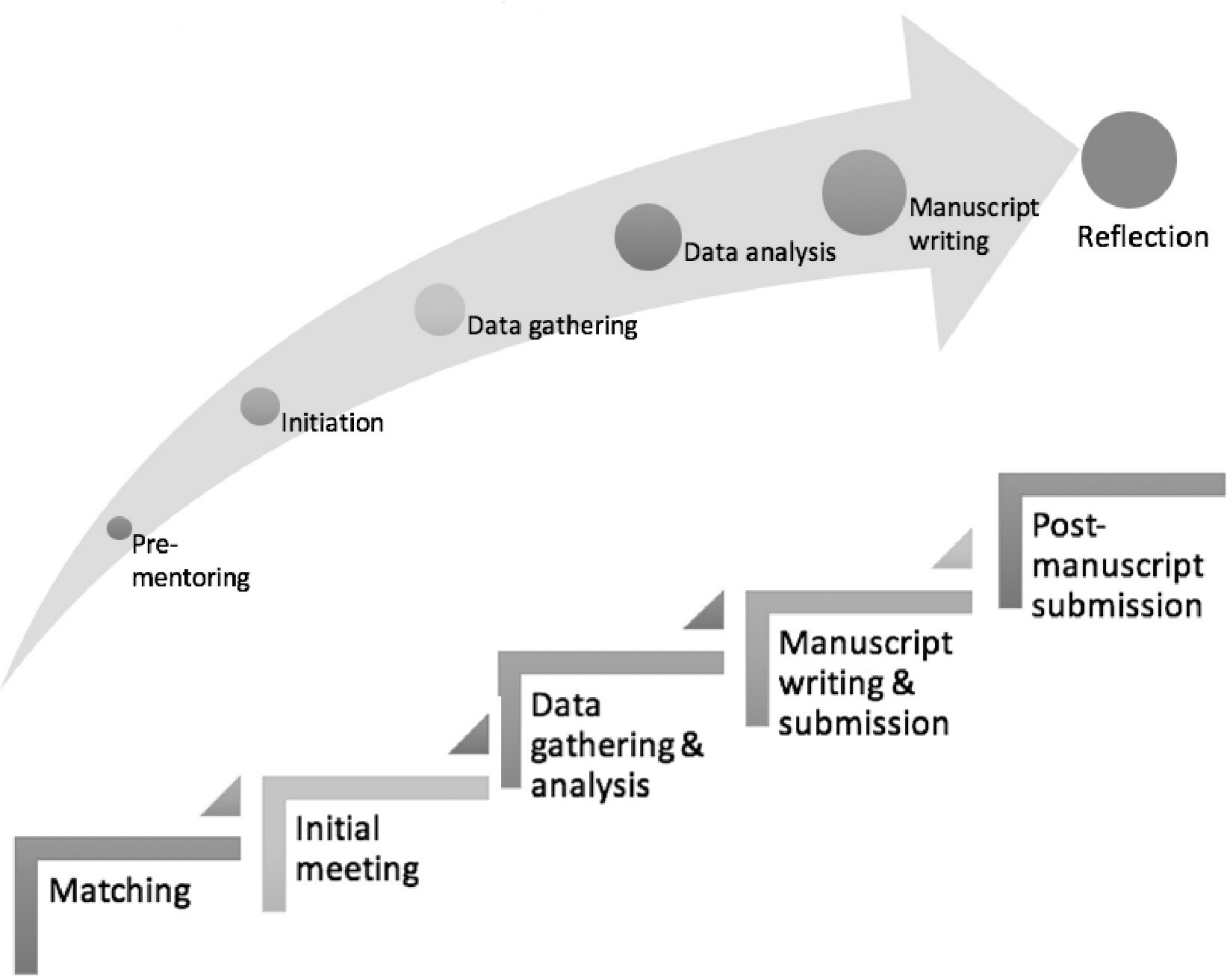
The Stages of mentoring.

The 2 themes identified in this study provide the first clinical evidence of mentoring’s nature proposed by recent reviews of mentoring practice in Palliative Medicine, Family Medicine, Internal Medicine, nursing, social work, occupational therapy and physiotherapy [7–11, 13, 15–17, 58, 71, 72]. The 6 stages of mentoring (mentoring stages) corroborate this data highlighting how the different phases of the research process are linked with different phases of the research process. It also highlights mentoring’s entwined nature with each mentoring stage cascading into the next, forming the basis for the stages that follows Fig 1.

Mentoring stages may be viewed as ‘circumscribed sequential projects’ with ‘specific goals and competency requirements’ that build on one another to achieve an overarching goal. Each stage requires the mentee to attain specific skills, insights and abilities, making progress from one mentoring stage to the next, highlighting a competency-based process. This is in stark contrast to time-based progress in mentoring and has a number of ramifications.

Competency-based mentoring stages are driven by assessments of a mentee’s progress, ability and motivation. Whilst use of assessments runs contrary to some prevailing mentoring theories a competency-based approach necessitates such evaluations the mentee’s and mentor’s psychosocial, academic, research and clinical situations and modifications in the host organization’s situation given their impact upon progress and attainment of in competencies [7–12, 14–17, 72, 79]. The PMI attempts to circumnavigate these concerns by ensuring that all assessments of the mentee’s progress are formative, holistic and longitudinal.

A personalized and competency-based approach to the PMI’s novice mentoring approach underscores its need to adapt its mentoring approach to accommodate to diverse influences upon the mentoring process without compromising mentoring experiences and breaching prevailing standards of practice and codes of conduct. Adaptability within the mentoring approach is evident at all mentoring stages, from the determination of specific goals and outcomes within agreed upon timelines and roles and responsibilities at the pre-mentoring stage to personalization’s of the initial research meeting, data gathering, review of findings and manuscript preparations stages. Adaptability within the PMI novice mentoring approach is also apparent in the characteristics of communication at different junctures of the mentoring process. Both examples highlight the influence of assessments in informing adaptations to the mentoring process.

Such adaptability however does not threaten the consistency and oversight of the PMI mentoring process courtesy of its structured research program. The presence of clear objectives and outcomes of the project and the agreed upon goals, roles and responsibilities, timelines and a code of conduct based upon professional and academic standards of practice ensures consistency in mentoring experiences, interactions, success and reflections.

## Limitations

Whilst an interview approach to appraising mentoring relationships and programs have been determined to be the gold standard, use of retrospective data at a single time point a variable distance from the end of the mentoring process invites recall bias [80]. The presence of single data points also limits the depth to which it is possible to delve into the various aspects of the mentoring process and the mentoring experiences.

The small sample size and a uniquely structured approach around a research process may limit the applicability of these findings in other settings.

## Conclusion

We believe that this study successfully answers the question “what are the experiences of mentees in a novice mentoring program?” providing new insights into mentoring experiences and process in a structured novice mentoring program that employs a consistent mentoring approach. This study shows mentoring to be a dynamic progressive process through which mentors advance through various stages to complete a research project. This study also found that communication is the main vehicle through which growth and maturation occurs.

Whilst there is more to be understood about the role of the mentor, it is clear that for mentors to be responsive, provide holistic and timely support and address the evolving needs of the mentee and the mentoring relationship, mentors must be trained to evaluate the mentoring relationship and the mentee’s needs and progress. Effective tools that move beyond prevailing reliance upon “*Cartesian reductionism and Newtonian principles of linearity*” [80] are required to provide longitudinal, multifaceted assessment tools.

In addition, the mentoring program should be guided by a balance between flexibility to meet the changing needs of the mentee, mentor, the host organization and their mentoring relationship and structure in the form of mentoring standards, codes of conduct and standards of practice that confines practice to acceptable parameters. Whilst the clearly demarcated research phases around which the PMI is created aids in ensuring that any changes to the mentoring program remains within the structure of the program, there is little reason to assume that similarly structured programs would not be equally successful in other clinical settings beyond the Palliative Medicine setting.

## Supporting information

S1 Table (XLSX)
